# Polychlorinated Biphenyl (PCB)-Degrading Potential of Microbes Present in a Cryoconite of Jamtalferner Glacier

**DOI:** 10.3389/fmicb.2017.01105

**Published:** 2017-06-15

**Authors:** Nancy Weiland-Bräuer, Martin A. Fischer, Karl-Werner Schramm, Ruth A. Schmitz

**Affiliations:** ^1^Institute for General Microbiology, Christian-Albrechts-Universität zu KielKiel, Germany; ^2^Molecular EXposomics, German Research Center for Environmental Health, Helmholtz Zentrum München GmbHNeuherberg, Germany

**Keywords:** cryoconite, microbial communities, biodegradation, polychlorinated biphenyls (PCBs), polycyclic aromatic hydrocarbon (PAH), organochlorine pesticide (OCP)

## Abstract

Aiming to comprehensively survey the potential pollution of an alpine cryoconite (Jamtalferner glacier, Austria), and its bacterial community structure along with its biodegrading potential, first chemical analyses of persistent organic pollutants, explicitly polychlorinated biphenyls (PCBs) and organochlorine pesticides (OCPs) as well as polycyclic aromatic hydrocarbons (PAHs), revealed a significant contamination. In total, 18 PCB congeners were detected by high resolution gas chromatography/mass spectrometry with a mean concentration of 0.8 ng/g dry weight; 16 PAHs with an average concentration of 1,400 ng/g; and 26 out of 29 OCPs with a mean concentration of 2.4 ng/g. Second, the microbial composition was studied using 16S amplicon sequencing. The analysis revealed high abundances of Proteobacteria (66%), the majority representing α-Proteobacteria (87%); as well as Cyanobacteria (32%), however high diversity was due to 11 low abundant phyla comprising 75 genera. Biodegrading potential of cryoconite bacteria was further analyzed using enrichment cultures (microcosms) with PCB mixture Aroclor 1242. 16S rDNA analysis taxonomically classified 37 different biofilm-forming and PCB-degrading bacteria, represented by *Pseudomonas*, *Shigella*, *Subtercola*, *Chitinophaga*, and *Janthinobacterium* species. Overall, the combination of culture-dependent and culture-independent methods identified degrading bacteria that can be potential candidates to develop novel bioremediation strategies.

## Introduction

Most of our biosphere is permanently cold (Morita, 1975; Russell et al., 1990; Margesin and Miteva, 2011). Among these cold environments, snow and glaciers permanently or seasonally cover up to 35% of the Earth’s terrestrial surface area (Miteva, 2008; Anesio and Laybourn-Parry, 2012). In areas of bare glacier ice, the most common water reservoirs are small supraglacial melt depressions known as “cryoconite holes” (Wharton et al., 1985; Southwell, 2014; Takeuchi, 2014). Cryoconites, continuously reported from glaciers worldwide, e.g., Alps, Arctic, Antarctic, Greenland, and Himalayas (Wharton et al., 1981; Kohshima, 1987; De Smet and Van Rompu, 1994; Margesin et al., 2002), cover up to 6% of the glacier surface (Fountain et al., 2004). Cryoconites are presumed to be “ice-cold hot-spots of microbial diversity and activity” (Edwards et al., 2013a) as they are the most biologically active habitats within glacial ecosystems (Säwström et al., 2002). Thus, they provide an ideal environment for a diverse variety of psychrophilic microorganisms, including bacteria, algae, viruses and yeasts (Margesin et al., 2002; Christner et al., 2003; Anesio et al., 2007). Until today, studying cryoconite microbial communities using molecular tools has mainly focused on polar regions (Christner et al., 2003; Foreman et al., 2007; Edwards et al., 2011, 2013a,b; Cameron et al., 2012a,b; Zarsky et al., 2013; Stibal et al., 2015), whereas generally less is known about the microbial diversity of cryoconites on alpine glaciers. Edwards et al. (2013a) reported on a metagenome assembly from cryoconites on the Rotmoosferner in the Austrian Alps, which was dominated by the bacterial phyla Proteobacteria, Bacteroidetes, and Actinobacteria followed by Cyanobacteria. In addition, further comparative studies revealed that cryoconite communities differ due to the properties of the cryoconite debris (Telling et al., 2012) and in relation to glacier-specific factors (Edwards et al., 2011, 2013a,b).

Furthermore, glacial ecosystems are subjected to the fallout and accumulation of black carbon (BC) as well as chemical pollutants, and are thus important indicators of pollution events. The presence of incorporated organic environmental toxins, so called persistent organic pollutants (POPs), such as polychlorinated biphenyls (PCBs) and organochlorine pesticides (OCPs) as well as polycyclic aromatic hydrocarbons (PAHs) is often associated to atmospheric deposition and transport path of air masses (Bizzotto et al., 2009). It was shown that BC offers the most important binding phases for PAHs and PCBs in the environment (Lohmann et al., 2005; Koelmans et al., 2006; Bond et al., 2013), thereby connecting the global cycle of POPs to that of BC. Many POPs and PAHs are carcinogenic and suspected to disturb the development of humans and animals (Koelmans et al., 2006). Additionally, they are long-lasting and can be transported via the atmosphere over long distances. The snowmelt transports these chemicals for years into glacier lakes, where they accumulated in the sediment. Thus, glaciers are secondary sources for re-entry of POPs in the environment (Villa et al., 2003, 2004, 2006). Chemical analyses detected concentrations of up to 43 ng/L for PCBs and up to 168 μg/L for PAHs in contaminated glacial areas (Cappa et al., 2014). From an ecological perspective, glaciers are low-carbon ecosystems, where most of the existing organic carbon is derived from allochthonous inputs (Stibal et al., 2008). Accumulation of organic pollutants can represent a relevant source of nutrients, and thus potentially a hotspot for hydrocarbon-degrading microorganisms (Megharaj et al., 2011). Hydrocarbon-degrading microorganisms comprise less than 0.1% of the microbial communities in uncontaminated environments. In contrast, these organisms can constitute over 90% of the viable microorganisms in polluted ecosystems (Atlas, 1981). Numerous studies have demonstrated the capacity of microorganisms to efficiently degrade a wide range of hydrocarbons, phenol, phenol-related compounds, and petroleum hydrocarbons (Aislabie et al., 1998; Bej et al., 2000; Baraniecki et al., 2002; Samanta et al., 2002; Bergauer et al., 2005; Margesin, 2007; Margesin et al., 2007; Stibal et al., 2012; Kuddus et al., 2013). Thus, bacteria thriving in cold environments, especially in the highly bioactive cryoconites, represent considerable candidates for low-temperature bioremediation besides their key ecological role for nutrient cycling (Margesin, 2007). The potential of such hydrocarbon-degrading microorganisms has already led to the development of bioremediation techniques for contaminated soil and water, but nowadays these procedures are still challenging (Dua et al., 2002).

The present study aimed to identify the microbial community structure of a cryoconite on the alpine Jamtalferner glacier in Austria by focusing on the presence of bacteria regardless of other microorganisms such as fungi and archaea. Analytic measurements of a broad spectrum of harmful compounds, namely PCBs, PAHs and OCPs, were examined to quantify the presence of organic contaminants in the cryoconite. A combination of culture-dependent and culture-independent methods was performed to identify potential biodegrading organisms, which might be relevant for the development of novel bioremediation strategies.

## Materials and Methods

### Study Site and Sampling

**FIGURE 1:**
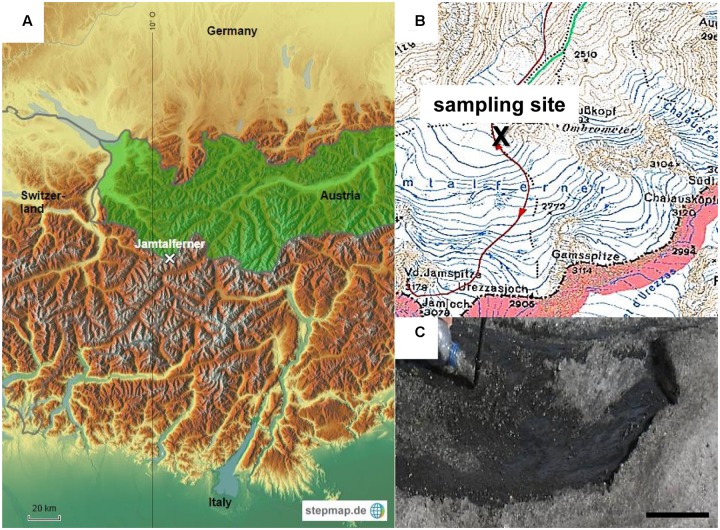
Sampling site. **(A)** Study location on glacier Jamtalferner in Austria. **(B)** Section of a compass map shows exact sampling site **(C)** Cryoconite on Jamtalferner, scale bar 8 cm.

Sampling was performed in September 2006 on Jamtalferner (47.51°N, 10.09°E), a medium sized valley glacier at the southern margin of Jam valley in the Silvretta group near the “Drei-Ländereck” in Austria. It covers an area of 3.5 km^2^ over a length of about 2.4 km. The lowest part of the glacier is located at an altitude of 2,420 m, the highest at Hintere Jamspitze (3,156 m). The cryoconite sample was collected near the glacier base at 2,700 m above sea level (see **Figure 1**) using a sterile 500 mL polyethylene terephthalate (PET) bottle and immediately transferred to the lab. The sample was aliquoted into 1 mL in polypropylene (PP) reaction tubes and frozen at -80°C. The sample contained both, cryoconite meltwater and detritus. All subsequent experiments mentioned below were performed within weeks after sampling.

### DNA Isolation

The cryoconite sample (10 g wet weight) was slowly thawed on ice before metagenomic DNA was extracted by direct lysis after a modified protocol by Henne et al. (1999) described in detail in Weiland et al. (2010) and Weiland-Bräuer et al. (2017). Total metagenomic DNA was further purified using a Qiagen-tip 100 (Qiagen Plasmid Midi Kit; Qiagen, Hilden, Germany). High-molecular-weight genomic DNA of enriched cultures was isolated from 5 mL overnight cultures using the AquaPure Genomic DNA Kit (Bio-Rad, Munich, Germany).

### 16S rRNA Gene Analysis

16S rRNA genes were PCR amplified from 10 ng isolated genomic DNA using the bacteria-specific primer 27F (5′-AGAGTTTGATCCTGGCTCAG-3′) and the universal primer 1492R (5′-GGTTACCTTGTTACGACTT-3′) (Lane, 1991), resulting in a 1.5 kbp PCR fragment. The fragment was cloned into pCRII-TOPO (Invitrogen, Karlsruhe, Germany). DNA sequences were determined by the sequencing facility at the Institute of Clinical Molecular Biology, University of Kiel, Kiel, Germany (IKM) using primer set 27F and 1492R or primer 27F singly.

DNA sequences were taxonomically classified using BLAST network service in the database of NCBI to determine their approximate phylogenetic affiliations. Tree reconstruction was performed with partial or full length 16S rRNA sequences using phylogeny.fr (Dereeper et al., 2008, 2010) with the Maximum-Likelihood method. Sequences were submitted to the NCBI database; accession numbers KT924431–KT924439 and KT931670–KT931706 (see Supplementary Table S2).

### 16S rRNA Gene Amplicon Sequencing

Primers used to construct the amplicon library were of the structure 5′-[Roche’s adaptor for long reads (Lib-L)] – [template-specific sequence]-3′. As template specific sequences, forward primer F338 (5′-ACTCCTACGGRAGGCAGCAG-3′) and reverse primer R802 (5′-TACNVGGGTATCTAATCC-3′) were used in a PCR amplification of the V3–V4 hypervariable region of the 16S rRNA gene (Dethlefsen et al., 2008; Claesson et al., 2010). Amplifications were conducted in two duplicate reactions of 50 μL, each containing 20 ng of template DNA and the Go*Taq* DNA Polymerase kit (Promega, Madison, WI, United States). Cycling conditions were: 94°C for 10 min, followed by 30 cycles of 94°C for 1 min, 44°C for 1 min and 72°C for 1 min, with a final step at 72°C for 10 min. The duplicate reactions were combined, amplicons size-checked and purified using MinElute Gel Extraction kit (Qiagen, Hilden, Germany). Purified amplicons were quantified using Quant-iT PicoGreen kit (Invitrogen, Darmstadt, Germany) and pooled to equimolar amounts. Pyrosequencing was carried out according to the manufacturer’s instructions using the GS FLX Titanium series kit (Sequencing Kit XLR70, Pico Titer Plate Kit 70 × 75, SV emPCR Kit/Lib-A, Maintenance Wash Kit; Roche, Mannheim, Germany) and 1/8 plate was sequenced on a Roche 454 GS-FLX Titanium platform at the IKM in 2007.

### Sequence Processing

Sequence processing was conducted with mothur v1.35.1 (Schloss et al., 2009) as recently described (Langfeldt et al., 2014; Weiland-Bräuer et al., 2015). Taxonomic classification was done using the ribosomal database project trainset release 14 (Cole et al., 2007, 2014). Bacterial sequences were further processed and binned to operational taxonomic units (OTUs) at a 97% similarity level. After the previous filtering and further quality steps, the analyzed cryoconite sample contained 15,479 high quality sequences belonging to 203 OTUs. Sequence data were deposited in the NCBI Sequence Read Archive (accession number PRJNA300438).

### Detection of *bphA* by PCR Amplification

The *bphA* gene encoding the large subunit of the iron–sulfur component of the key enzyme biphenyl dioxygenase for degradation of PCBs was PCR amplified using 10 ng isolated metagenomic DNA as well as genomic DNA of *Pseudomonas* spp. isolated from microcosms and the Go*Taq* DNA Polymerase kit (Promega, Madison, WI, United States) in 25 μL total volume. Primers for amplification of the 211 bp universal *bphA* fragment (Uhlik et al., 2007) and the 1,100 bp *bphA* gene of five PCB degrading groups (Sanseverino et al., 2002) were used for amplification (see Supplementary Table S1). PCB degrading bacteria in the cryoconite were determined based on gel electrophoresis, followed by sequencing of the PCR products at the sequencing facility at IKM.

### Isolation of Bacteria

#### Enrichment of Bacteria in Nutrient Broth

Bacteria were isolated from the cryoconite sample by streaking 100 μl of the original cryoconite sample and respective serial dilutions (10^-1^ to 10^-4^) on Nutrient Broth (NB, Carl Roth; Karlsruhe, Germany) agar plates, which were incubated at 4, 20, 30, and 37°C for at least 2 days. Obtained colonies were purified at least three times by repeated streaking. Pure cultures were analyzed for 16S rRNA genes and taxonomically classified via BLAST (Basic Local Alignment Search Tool) search using the National Center for Biotechnology Information (NCBI) database.

#### Enrichment Using Microcosms

Enrichment of bacteria on PCB using microcosms was performed according to Macedo et al. (2005) (Supplementary Figure S1). Droplets (2 μL) of Aroclor 1242 (50 mg/kg in transformer oil; Sigma-Aldrich, Munich, Germany) were placed on sterile standard borosilicate glass cover slips (24 mm × 60 mm; thickness, 0.17 mm, Carl Roth, Karlsruhe, Germany). The slides bearing 10 droplets of PCB mixture Aroclor 1242 were placed with the PCB droplets downward on the water surface of a reservoir filled with 20 mL sterile tap water and 20 g (2 mL) cryoconite sample. Three enrichments were started in parallel. The microcosms were kept at 4°C without agitation for 28 days. After 7, 14, and 28 days, three droplets were used for confocal laser scanning microscopy (CLSM) to detect biofilm formation on PCB droplets. In addition, after 28 days, six droplets were used for DNA isolation and the composition of the bacterial community enriched on PCB was analyzed by 16S rRNA gene analysis using the universal bacterial 16S primer pair 27F and 1492R, cloning and sequencing by Sanger sequencing.

### Microscopic Analysis

Light microscopy of the cryoconite sample was performed with an Axio Scope microscope and Axio Vision software (Zeiss, Jena, Germany). Biofilm formation on PCB droplets of microcosms was monitored after 7, 14, and 28 days of incubation. PCB droplets on the slides were stained with Nile Red for 15 min according to the manufacturer (Sigma-Aldrich, Karlsruhe, Germany). The sample was carefully rinsed twice with MilliQ water and counterstained using the nucleic acid-specific stain Syto9 (Invitrogen, Darmstadt, Germany). Samples were incubated for 15 min at RT. The entire three-dimensional structure of the PCB community was recorded by scanning along depth using a TCS SP CLSM (Leica, Wetzlar, Germany) and recording the stacks of cross sections simultaneously at the corresponding excitation wavelengths. The following settings were used for excitation and recording of emission signals, respectively: Nile Red, 488 and 550 to 700 nm; Syto9, 488 and 500 to 540 nm. For each field of view, an appropriate number of optical slices were acquired with a Z-step of 1 μm. Digital image acquisition, post-processing, analysis of the CLSM optical thin sections and three-dimensional reconstructions were conducted with the corresponding Leica software (provided for the TCS SP CLSM).

### Chemical Analysis of Hydrocarbon Contaminants

The contamination of the cryoconite sample with PCBs, PAHs, and OCPs was analyzed using high resolution gas chromatography/high resolution mass spectrometry (HRGC/HRMS). The executing laboratory is accredited for the analysis of PAHs, PCBs, and OCPs in various matrices (D-PL-14138-02-00). First, a dry matter determination of the cryoconite sample was performed that yielded 1.28 g. The sample was transferred to hydromatrix with 1 mL double-distilled water and rinsed three times with 1 mL double-distilled water followed by extraction using ASE 200. The hydromatrix mixture was filled in a 33 mL extraction cell added with isotopically labeled quantification standards listed in Supplementary Table S3. The cell was extracted with a solvent mixture of *n*-hexane/acetone (75:25, v:v) at 120°C and 12 MPa. Two static cycles of 10 min were sufficient for complete extraction. Water was removed using anhydrous sodium sulfate. The volume of the extract was reduced to 1 mL for further cleanup using a rotary evaporator.

A chromatography column was filled from bottom to top with 10 g of silica gel (LGC Standards, Wesel, Germany), 5 g Alumina B (with 3% water added) (LGC Standards, Wesel, Germany) and 2 g of anhydrous sodium sulfate. The column was washed prior to the sample addition with 60 mL of hexane/dichloromethane (1:1, v:v). 1 mL of sample extract was applied to the upper layer of sodium sulfate. Compounds were eluted with 100 mL *n*-hexane/dichloromethane (1:1, v:v) and again concentrated to 1 mL. A glass fiber filter was placed in the lower end of an empty 8 mL SPE glass cartridge. 1 g C18-modified silica gel (Chromabond C18, Macherey-Nagel, Düren, Germany) was added and covered with a further glass fiber filter. The SPE cartridge was conditioned with 5 mL acetonitrile under vacuum. Before sample application, a solvent change was carried out with 0.5 mL acetonitrile under a stream of nitrogen and topped on the SPE. Following, 5 mL acetonitrile was eluting the analytes from the SPE cartridge and concentrated to 0.2–0.3 mL under nitrogen stream. The final eluate was transferred into a vial, where it was reduced to a final volume of 20 μL already containing the respective recovery standards (see Supplementary Table S4). The analytical measurement of PCBs, PAHs, and OCPs potentially present in the analyzed cryoconite sample was determined by HRGC/HRMS. The parameters used for analytical measurements are listed in Supplementary Table S5. In addition to the cryoconite sample, the PET sampling bottle as well as a PP storage tube (see “Study site and sampling”) were analyzed regarding the presence of PCBs, PAHs and OCPs. Therefore, bottle and storage tubes were rinsed with *n*-hexane/acetone (75:25, v:v), followed by HRGC/HRMS measurement of the solvent.

## Results

### Persistent Organic Pollutants in the Alpine Cryoconite

Chemical analyses of a broad spectrum of potentially harmful, organic substances were performed using HRGC coupled to isotope-dilution HRMS in order to identify and quantify chemical pollutants in the cryoconite sample from Jamtalferner glacier. In total, samples were assayed for 18 PCB congeners, 16 PAHs and 29 OCPs (**Tables 1**–**3**). All analyzed PCB congeners were detected in the cryoconite with concentrations ranging from 12 pg/g dry weight (PCB #81) to 3847 pg/g (PCB #138) (**Table 1**). Concentrations of PAHs were one magnitude higher as PCB quantities; with a minimum of 44 ng/g Acenaphthene and a maximum of 6908 ng/g Fluoranthene (**Table 2**). All analyzed PAHs were detected in high amounts. Furthermore, 26 out of 29 OCPs were detected in the cryoconite with a notable maximum concentration of 38 ng/g for 4,4′-DDT (**Table 3**). The additionally analyzed PET sampling bottle as well as the PP storage tubes (see Materials and Methods) did not showed atypical concentrations of PCBs, PAHs, and OCPs (**Tables 1**–**3**). To sum up, a significant contamination with a broad spectrum of organic environmental toxins was observed in the surface of the cryoconite on Jamtalferner glacier.

**Table 1:** Concentrations of polychlorobiphenyls (PCBs) in the cryoconite from Jamtalferner.

Polychlorobiphenyl (PCB)	Concentration (pg/g dry weight) in sample	Concentration (pg/g dry weight) in PET bottle	Concentration (pg/g dry weight) in PP tube
Indicator PCB (congener)			
2,4,4′-Trichlorobiphenyl (28)	252	98	36
2,2′,5,5′-Tetrachlorobiphenyl (52)	339	138	62
2,2′,4,5,5′-Pentachlorobiphenyl (101)	1115	180	97
2,2′,3,4,4′,5′-Hexachlorobiphenyl (138)	3847	176	159
2,2′,4,4′,5,5′-Hexachlorobiphenyl (153)	3629	185	140
2,2′,3,4,4′,5,5′-Heptachlorobiphenyl (180)	2489	80	71
Non-ortho PCB (congener)			
3,3′,4,4′-Tetrachlorobiphenyl (77)	205	8	4
3,4,4′,5-Tetrachlorobiphenyl (81)	12	n.d.	n.d
3,3′,4,4′,5-Pentachlorobiphenyl (126)	76	n.d.	n.d
3,3′,4,4′,5,5′-Hexachlorobiphenyl (169)	16	n.d.	n.d
Mono-ortho PCB (congener)			
2,3,3′,4,4′-Pentachlorobiphenyl (105)	504	38	31
2,3,4,4′,5-Pentachlorobiphenyl (114)	21	n.d.	n.d.
2,3′,4,4′,5-Pentachlorobiphenyl (118)	1210	86	76
2′,3,4,4′,5-Pentachlorobiphenyl (123)	27	n.d.	n.d.
2,3,3′,4,4′,5-Hexachlorobiphenyl (156)	291	15	16
2,3,3′,4,4′,5′-Hexachlorobiphenyl (157)	83	n.d.	n.d.
2,3′,4,4′,5,5′-Hexachlorobiphenyl (167)	204	12	6
2,3,3′,4,4′,5,5′-Heptachlorobiphenyl (189)	66	n.d.	n.d.


*n.d., not detected.*

**Table 2:** Concentrations of polycyclic aromatic hydrocarbons (PAHs) in the cryoconite.

Polycyclic aromatic hydrocarbon (PAH)	Concentration (ng/g dry weight) in sample	Concentration (ng/g dry weight) in PET bottle	Concentration (ng/g dry weight) in PP tube
Naphtalene	953	1.7	n.d.
Acenaphthylene	122	0.4	0.3
Acenaphthene	44	0.3	0.2
Fluorene	314	0.8	0.5
Phenanthrene	3351	8.3	3.2
Anthracene	259	0.5	0.7
Fluoranthene	6908	12.0	2.4
Pyrene	3878	9.9	1.9
Benzo(a)anthracene	707	2.9	0.6
Chrysene	1824	7.1	1.3
Beno(b)fluoranthene	1241	9.4	1.2
Benzo(k)fluoranthene	394	4.2	0.5
Benzo(a)pyrene	438	5.3	0.5
Indeno(1,2,3-c,d)pyrene	946	7.3	0.6
Benzo(g,h,i)perylene	967	7.5	0.6
Dibenzo(a,h)anthracene	92	1.1	0.1


*n.d., not detected.*

**Table 3:** Concentrations of organochlorine pesticides in cryoconite.

Organochlorine pesticides	Concentration (pg/g dry weight) in sample	Concentration (pg/g dry weight) in PET bottle	Concentration (pg/g dry weight) in PP tube
1,2,3,4,5,6-Hexachlorocyclohexanes (HCH)			
α-HCH	2729	12	7
β-HCH	232	10	20
γ-HCH	1816	88	39
δ-HCH	396	15	20
ε-HCH	58	23	n.d.
Benzene derivatives			
Pentachlorobenzene	996	18	17
Hexachlorobenzene	2199	155	119
Pentachloroanisole	214	41	21
Octachlorostyrene	169	20	9
Dichlorodiphenylethanes (DDTs) pesticides			
4,4′-Dichlorodiphenyltrichloroethane (4,4′-DDT)	37812	218	71
2,4′-Dichlorodiphenyltrichloroethane (2,4′-DDT)	5593	73	29
4,4′-Dichlorodiphenyldichloroethane (4,4′-DDD)	4672	37	6
2,4′-Dichlorodiphenyldichloroethane (2,4′-DDD)	1013	16	5
4,4′-Dichlorodiphenyldichloroethene (4,4′-DDE)	7132	187	49
2,4′-Dichlorodiphenyldichloroethene (2,4′-DDE)	794	23	11
Chlordane pesticides			
*trans*-Chlordane	937	n.d.	n.d.
*cis*-Chlordane	369	n.d.	n.d.
*oxy*-Chlordane	21	n.d.	n.d.
Heptachlor	n.d.	5	n.d.
*cis*-Heptachloroepoxide	93	4	6
*trans*-Heptachloroepoxide	n.d.	n.d.	n.d.
Aldrin	n.d.	n.d.	n.d.
Dieldrin	938	24	24
Endrin	142	n.d.	14
Endosulfan-I	685	81	n.d.
Endosulfan-II	1006	n.d.	86
Endosulfan-sulfate	411	6	7
Methoxychlor	61	9	21
Mirex	54	26	27


*n.d, not detected.*

### Bacterial Community Structure of a Cryoconite on Jamtalferner Glacier

**FIGURE 2:**
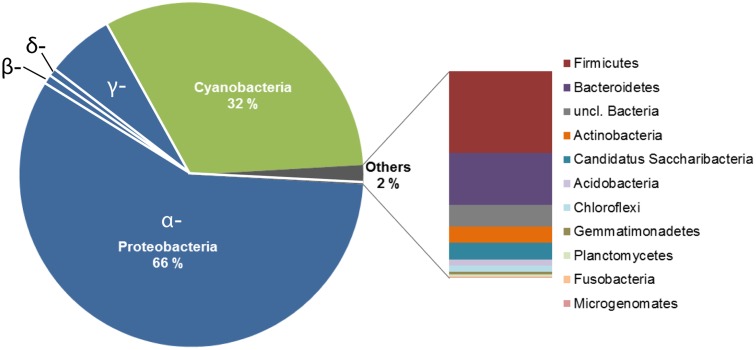
Deep sequencing analysis of the bacterial communities in the alpine cryoconite. The community composition of the cryoconite on Jamtalferner was analyzed using 454 deep sequencing technology (Illumina, Roche) of the V3–V4 region of the 16S rRNA gene. The cryoconite composition is displayed at bacterial phylum level.

**FIGURE 3:**
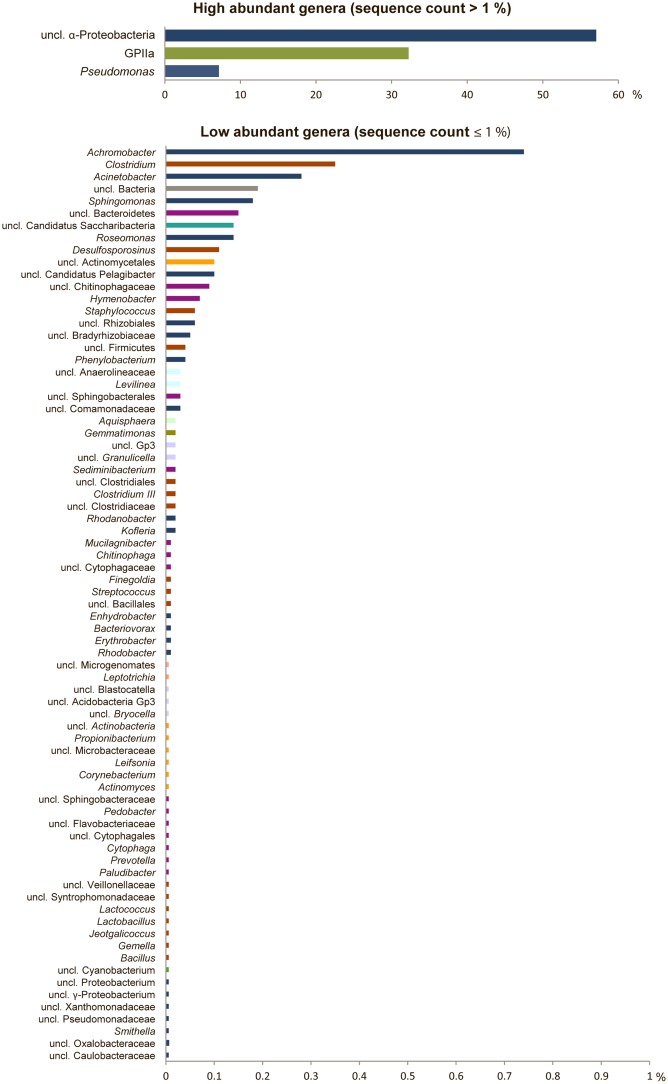
High bacterial diversity in the alpine cryoconite. Bacterial community composition of the cryoconite displayed at genus level for high abundant (**upper**) and low abundant genera (**lower**). Color code reflects phylum affiliation.

The bacterial community structure of the upper surface of an alpine cryoconite from Jamtalferner glacier was determined by 454 pyrosequencing using the hypervariable regions V3–V4 of the 16S rRNA gene. 15,479 sequences were included in the 16S amplicon analysis after processing and resulted in the identification of 203 different OTUs (97% sequence similarity) (Supplementary Table S6). It was observed that the microbial community was dominated by the phyla Proteobacteria (66%) and Cyanobacteria (32%). Within the most dominant bacterial phyla, a number of classes were present. Of particular mention is the abundance of α-Proteobacteria, which accounted for 87% of all Proteobacteria, with γ-Proteobacteria (11%) and other classes (β-Proteobacteria, 1%; δ- Proteobacteria, 0.06%; unclassified Proteobacteria, 0.01%) accounting for the remainders. However, the observed high diversity of the community was reflected by 11 other phyla present in low abundances, namely Firmicutes, Bacteroidetes, Actinobacteria, Candidatus Saccharibacteria, Acidobacteria, Chloroflexi, Gemmatimonadetes, Planctomycetes, Fusobacteria, Microgenomates (**Figure 2**). The relative distribution of bacterial genera is depicted in **Figure 3**. High abundant genera were unclassified α-Proteobacteria (57%), followed by genus GpIIa belonging to phylum Cyanobacteria (32%) and γ-Proteobacterium *Pseudomonas* (7%). However, these three genera included only 13 OTUs, whereas 190 OTUs were present within the 75 low abundant genera (**Figure 3** and Supplementary Table S6). Overall, the community structure of the cryoconite was characterized by high bacterial diversity including both, heterotrophic and phototrophic bacteria.

### Enrichment of Cryoconite Bacteria

**FIGURE 4:**
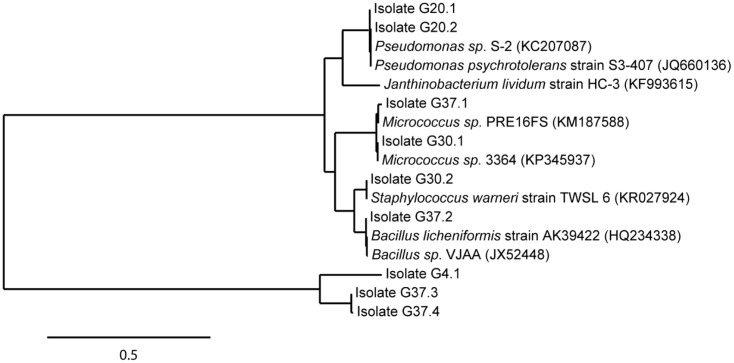
Phylogenetic position of identified isolates enriched from the cryoconite. Isolates (in total 9) were enriched from the cryoconite ample on Nutrient Broth medium at 4, 20, 30, and 37°C. The tree was calculated from full-length 16S rRNA gene sequences by the Maximum-Liklihood method. Nearest relatives to the 16S rRNA sequences of isolates were obtained via BLAST search using the NCBI database. The scale bar represents evolutionary distance (substitutions per nucleotide).

A total of 29 cryoconite bacteria were isolated by enrichment on rich medium (NB agar plates) incubated at various temperatures (4, 20, 30, and 37°C). Isolates from different agar plates varying in morphology (shape, color) were verified by several streaks for single clones resulting in 17 taxonomically classified isolates. Sequence analysis identified nine unique isolates based on 99% identity on nucleotide level. All isolates were identified at least at genus level. As illustrated in the phylogenetic tree (**Figure 4**), Gram-positives were represented by *Micrococcus* (G30.1, Accession No. KT924434; G37.1, Accession No. KT924436), *Staphylococcus* (G30.2, Accession No. KT924435), and *Bacillus* species (G37.2, Accession No. KT924437; G37.3, Accession No. KT924438; G37.4, Accession No. KT924439). Gram-negative bacteria were represented by species of the genera *Janthinobacterium* (G4.1, Accession No. KT924431) and *Pseudomonas* (G20.1, Accession No. KT924432; G20.2, Accession No. KT924433). Pertaining to the deep sequencing analysis, almost all NB enriched bacteria detected belonged to the low abundant taxa and their 16S rRNA sequences matched with certain OTUs listed in Supplementary Table S6 (see Supplementary Table S7).

### Biodegrading Potential of Cold-Adapted Bacteria from Cryoconite

Based on the heavy contamination detected on the cryoconite and results of 16S amplicon sequencing, we assumed increased occurrence of biodegrading bacteria in the sample. For identification of remediating bacteria, we experimentally studied solely the degradation of PCBs by a combination of two approaches (i) a metagenomic and (ii) an enrichment-driven approach.

**FIGURE 5:**
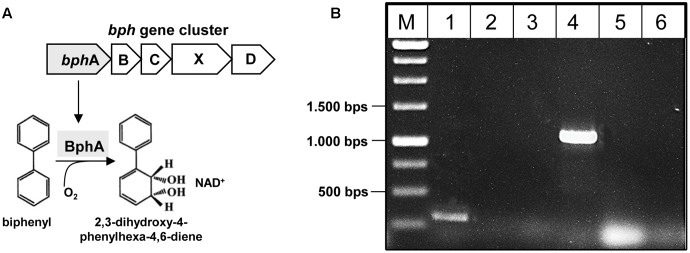
Detection of *bphA* in the metagenome of the cryoconite. **(A)** Biphenyl-degrading microorganisms are able to degrade several PCB congeners using the same enzymatic system. The catabolic enzymes involved in the biodegradation of PCBs are organized in the *bph*ABCXD operon where the biphenyl dioxygenase BphA is the first key enzyme. **(B)** Detection of *bphA* using universal primer bphA 463/674 (lane 1), and specific primer sets for PCB Gram- group1 (lane 2), PCB Gram- group2 (lane 3), PCB Gram- group3 (lane 4), PCB Rhodo group1 (lane 5), PCB Rhodo group2 (lane 6) (see also Supplementary Table S1).

First, the presence of the key gene *bphA* of the PCB degradation process (encoding the large subunit of biphenyl dioxygenase) was monitored in the metagenomic DNA by PCR amplification (**Figure 5A**). The 211 bp *bphA* fragment was successfully amplified in the cryoconite metagenome using universal primers (**Figure 5B**, lane 1). In addition, using taxa-specific primers resulted in the amplification of the 1,100 bp *bphA* fragment exclusively for taxa group “Gram-negative 3” containing *Pseudomonas* and *Ralstonia* species (**Figure 5B**, lane 4). Sequence analysis of the cloned 1,100 bp *bphA* fragment verified the presence of the key gene and enabled classification to *Pseudomonas* species.

**FIGURE 6:**
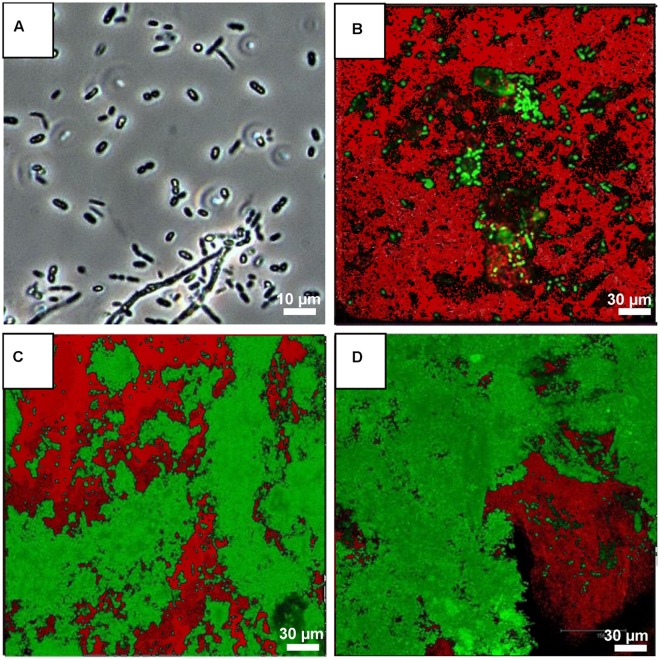
Biofilm formation on PCB droplets over time. **(A)** Light microscope image of the bacterial community of the cryoconite sample. Confocal laser scanning images (CLSMs) demonstrating biofilm formation on PCB droplets over time (**B**, 7 days; **C**, 14 days; and **D**, 28 days of incubation at 4°C). Images are overlays of Syto9 signals of living PCB-degrading bacterial cells (green), and Nile red signals of PCB (Aroclor 1242; red).

Second, floating dish microcosm experiments were performed to enrich potentially present PCB-degrading bacteria (Supplementary Figure S1). The microcosms were kept at 4°C without agitation for 28 days. After 7, 14, and 28 days, three droplets were analyzed by CLSM to detect biofilm formation on PCB droplets. **Figure 6** depicts the formation and development of a bacterial biofilm on PCB droplets. Initially, bacterial cells colonized the glass substratum of the slide within 3 days. Single cells and cell aggregates were detected on the glass substratum close to the PCB droplet, but almost no cells were observed on the droplet. After 7 days, first bacterial cells populated the PCB droplets. Within the next 21 days of incubation, large microbial aggregates were observed on the PCB surface as depicted in **Figures 6C,D**. Bacterial cells formed aggregates on the PCB droplets, which matured to compact biofilms. After 28 days of incubation, degradation of PCBs was indicated by observing perforated PCB droplets (**Figure 6D**).

**FIGURE 7:**
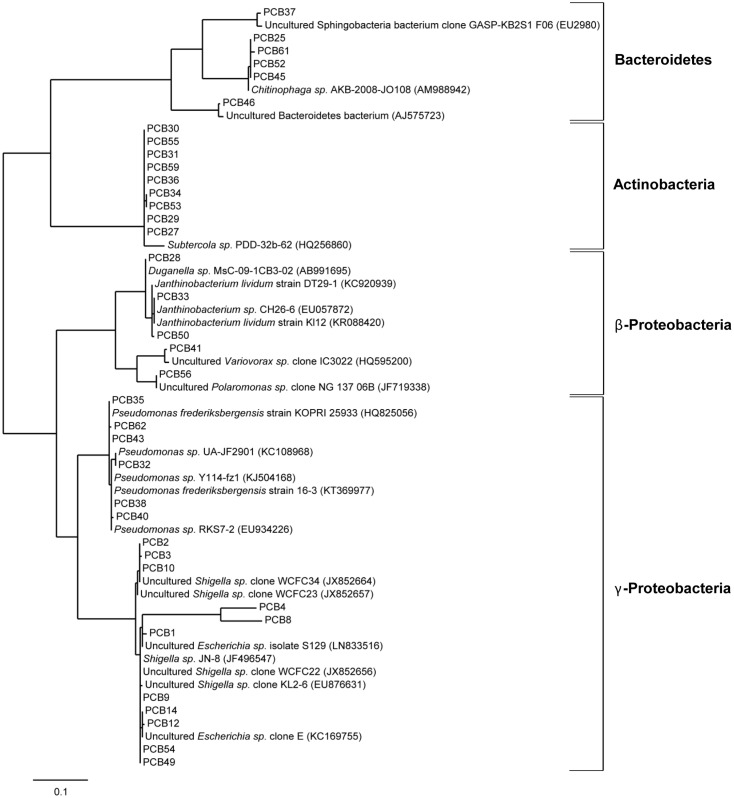
Phylogenetic analysis of cold-adapted PCB degraders enriched in microcosms. Phylogenetic tree of 37 bacterial clones enriched from the cryoconite on PCB droplets in microcosms including four identified taxonomic groups. The tree was calculated from partial 16S rRNA gene sequences by the Maximum-Liklihood method 16S rRNA sequences of isolates (PCB1-PCB62) are represented with their nearest relatives obtained via BLAST search using the NCBI database. The scale bar represents evolutionary distance (substitutions per nucleotide).

To verify the enrichment of bacteria on PCB after 28 days of incubation, six droplets were pooled and used for DNA isolation, followed by 16S rRNA analysis using the universal bacterial primer set 27F and 1492 followed by cloning and Sanger sequencing (see Materials and Methods). Sequence analysis using the NCBI BLAST tool and the multiple sequence alignment tool CLUSTALW resulted in identification of 37 unique sequences (based on 99% identity on nucleotide level) out of a total of 188. The taxonomical classification resulted in the assignment of these sequences to three different phyla Bacteroidetes, Actinobacteria, and Proteobacteria; the latter subdivided into β- and γ-Proteobacteria (**Figure 7**). Within the phylum Bacteroidetes five sequences were classified to class Sphingobacteriia and genus *Chitinophaga*. Actinobacteria were exclusively represented by Gram-positive *Subtercola* species of family Microbacteraceae. Within the class of β-Proteobacteria seven sequences were assigned to genera *Duganella*, *Janthinobacterium*, *Polaromonas* and *Variovorax*, whereas *Shigella*, *Escherichia*, and *Pseudomonas* were identified within γ-Proteobacteria. Additionally, isolates taxonomically classified to *Pseudomonas* spp. were analyzed regarding the presence of the *bphA* gene resulting in identification of the respective gene in isolates PCB32, PCB38, and PCB40 (see **Figure 7**). Again, almost all enriched bacteria were assigned to the low abundant taxa, except *Pseudomonas*. 16S sequences of isolates obtained from the Sanger sequencing approach matched with respective OTUs assessed by amplicon sequencing (Supplementary Table S7).

## Discussion

### Heavy Contamination of the Alpine Cryoconite with POPs and PAHs

Since the late 1960s detectable concentrations of POPs and PAHs have been discovered in many environmental matrices, e.g., air, water, and sediments in the Alps, Antarctic, and Arctic (Halsall, 2004; Bassan et al., 2005; Tremolada et al., 2008; Fuoco et al., 2009; Hansen et al., 2014; Pisso et al., 2014; Vecchiato et al., 2015). It is already known that their long range transport and bioaccumulation significantly impacts human health and the environment, thus POP and PAH exposure is supposed to cause developmental defects, chronic illnesses, cancer, and death (Kageson, 1998). Particularly, glaciers have been shown to be secondary emission sources by re-emitting POPs long after atmospheric deposition (Kallenborn et al., 2012), thus the identification and quantification of POPs and PAHs in glaciers is important for the prediction of secondary emission events and corresponding contamination scenarios. So far, chemical detection of POPs and PAHs in cold environments was mostly conducted in glacial ice and sediment cores (Villa et al., 2006; Bizzotto et al., 2009; Nadal et al., 2015). To the best of our knowledge, this is the first study presenting detailed chemical analyses of PCBs, PAHs, and OCPs in a cryoconite.

Almost all POPs identified in the cryoconite from Jamtalferner, including a broad range of PCB congeners and various OCPs, are listed in the Stockholm Convention, a global treaty to protect human health and the environment from these chemicals (Stockholm Convention, 2009). In general, concentrations of POPs and PAHs vary between study sites in the Alps, Arctic, and Antarctic as well as between different altitudes and nutrient sources comprising xenobiotic entry (Bradley et al., 2014). Documented PCB concentrations in pristine glaciers are on average 0.5 ng/L (Bogdal et al., 2009), whereas PCB concentrations in contaminated glacier areas were approximately 20 ng/L (Cappa et al., 2014); and sediment cores even range from 2 to 132,000 ng/g dry weight (Bigus et al., 2013). These reference data already show that a comparison between several studies is difficult because of different measurement methods and units specified for POP and PAH concentrations. Assuming that one kilogram of dry matter as specified for sediments or debris-associated POP/PAH concentrations is equivalent to 1 lt sample volume as indicated for snow, melt water, and ice cores; an approximate comparison can be drawn. Taking this assumption into account, for instance the average concentration of PCBs in the analyzed cryoconite is two magnitudes of order higher than in contaminated glacier areas with up to 3.8 ng/g dry weight (PCB #138, see **Table 1**). Similar high concentrations were also revealed for PAH and OCP concentrations (**Tables 2**, **3**). In comparison with reference data, our observed PAH and OCP concentrations are similar to those in heavily polluted regions (Blais et al., 2001; Villa et al., 2006; Bigus et al., 2013; Cappa et al., 2014). All those comparisons clearly show the heavy contamination of the analyzed sample from the Jamtalferner cryoconite with a broad range of environmental toxins. It might be suggested that the BC particles act as sorbent for the pollutants leading to detected high concentrations of PCBs, PAHs, and OCPs. Those pollutants can strongly force for the selection of biodegrading bacteria in generally carbon-poor environments, and thus provide potential to develop remediation strategies with so far unknown biodegrading bacteria.

### Highly Diverse Bacterial Community in the Cryoconite from Jamtalferner Glacier

In the meantime, the diversity of microbial communities present in cold environments is well-known (Carpenter et al., 2000; Amato et al., 2007; Simon et al., 2009; Larose et al., 2010; Harding et al., 2011; Gutiérrez et al., 2015). However, previous analyses have primarily focused on culture- and microscopy-based approaches (Margesin et al., 2002, 2007; Edwards et al., 2013b; Singh et al., 2014). As expected, many of the bacteria found in the cryoconite from Jamtalferner in this study are closely related to bacteria obtained from permanently cold environments such as alpine lakes, Antarctic sea ice, and freshwater lakes (Morita, 1975; Margesin et al., 2007; Bowman, 2014). Taxonomical classification revealed that the analyzed cryoconite mainly consisted of Proteobacteria and Cyanobacteria. Within these phyla, Proteobacteria were dominated by α-Proteobacteria; Cyanobacteria exclusively contained genus GpIIa including *Prochlorococcus* and *Synechococcus*. The latter are both known as the most important CO_2_ fixing bacteria on earth (Bryant and Frigaard, 2006) arguing that they are one of the primary producers in the cryoconite. Several other studies also revealed the predominance of proteobacterial lineages within bacterial cryoconite communities (Edwards et al., 2011, 2013a; Cameron et al., 2012b; Franzetti et al., 2013; Zarsky et al., 2013). It is suggested that Proteobacteria are well-adapted to respond to regular environmental variations typical for short active summer seasons in cold environments (Edwards et al., 2014). A key strategy for survival in such cold environments appears to be the extremely efficient scavenging and recycling of nutrients, as demonstrated for the Proteobacteria-dominated ice-shelf microbial mat metagenomes (Varin et al., 2010, 2012). It is possible that Proteobacteria, and in particular α-Proteobacteria, be important players within the Alp cryoconite community. In addition, the identified predominance of Proteobacteria over Cyanobacteria challenges the assumption that Cyanobacteria are the exclusive contributors to primary production within cryoconites (Edwards et al., 2011, 2014). Photosynthetic Proteobacteria, such as *Rhodobacter* and *Erythrobacter*, were present in the sequencing data as well as other phototrophic taxa like *Gemmatimonas* and *Chloroflexi*. In conclusion, our data demonstrate that a diverse microbial community is present in the cryoconite from Jamtalferner that are well-known in supraglacial environments.

### Remediation of PCB by Cold-Adapted Bacteria toward Potential Application

Nowadays, polluted glaciers are considered as the ultimate sink for many POPs (Blais et al., 2001). The breakdown of these hazardous substances into less toxic or non-toxic substances in such an environment could prevent the entry of POPs into the groundwater and biota, and ultimately avoid their accumulation in animals and humans. Currently, bioremediation is accepted as the practicable method to eliminate POPs as well as PAHs from the environment, because of its advantages over other processes as landfill, soil washing, and incineration (Bajaj and Singh, 2015). However, less information is available on the biodegradation of POPs and PAHs in contaminated cold environments, but it is known that low temperatures affect the rate of biodegradation due to adaptation of physical parameters of the contaminants such as increased viscosity, decreased volatilization and reduced bioavailability (Margesin, 2007; Margesin et al., 2007). In addition, cold conditions also influence microbial activity by reducing metabolic turnover rates (Feller, 2010), and thus make bioremediation in remote cold areas more difficult (Bej et al., 2009). Several reports showed the capability of cold-adapted bacteria to biodegrade POPs/PAHs and their metabolic products under low temperature conditions (Jones and De Voogt, 1999; Bergauer et al., 2005; Hesselsoe et al., 2005; Haritash and Kaushik, 2009; Bajaj and Singh, 2015). Cryoconites as important supraglacial niches and microbial hot-spots of diversity and activity are therefore an ideal habitat, where cold-adapted bacteria with remediation potential can be found. The detection of the gene fragment encoding for biphenyl dioxygenase, involved in the aerobic degradation of PCBs, experimentally identified *Pseudomonas*, one of the major genera in the cryoconite, as potential PCB-degrader. Lastly, the successful enrichment of bacteria in Aroclor 1242 microcosms assigned to genera *Pseudomonas*, *Shigella*, *Polaromonas*, *Variovorax*, *Janthinobacterium*, *Subtercola*, and *Chitinophaga* as well as the corresponding depletion of PCBs reflected by CLSM imaging indeed demonstrated PCB degradation by cryoconite embedded bacteria. Almost all of the 37 microcosm-enriched bacteria were identified by phylogenetic analyses as close relatives to known biodegraders (see **Table 4**).

**Table 4:** List of selected bacteria identified in the cryoconite, which are potentially able to biodegrade hydrocarbons.

Hydrocarbon degrading bacterium		Reference
	
Phylum, class, or candidate division	Genus	
Actinobacteria	*Micrococcus*	Li et al., 2005; Sharma et al., 2010; Gallego et al., 2014; Su et al., 2015
	*Subtercola*	Singh et al., 2014
Firmicutes	*Bacillus*	Margesin and Schinner, 2001; Margesin et al., 2003a; Lu et al., 2011; Lovecka et al., 2015
	*Clostridium*	MacRae et al., 1969; Sethunathan and Yoshida, 1973; Hou and Dutta, 2000
	*Desulfosporosinus*	Robertson et al., 2000; Sun et al., 2014
	*Staphylococcus*	Leaes et al., 2006; Mallick et al., 2007
Gemmatimonadetes	*Gemmatimonas*	Uhlik et al., 2012
α-Proteobacteria	*Bradyrhizobium*	Samanta et al., 2002; Macedo et al., 2005
	*Sphingomonas*	Zylstra and Kim, 1997; Aislabie et al., 1998; Saul et al., 2005
β-Proteobacteria	*Achromobacter*	Ahmed and Focht, 1973; Bacosa et al., 2012; Deng et al., 2014
	*Duganella*	Mera and Iwasaki, 2007
	*Janthinobacterium*	Hesselsoe et al., 2005
	*Polaromonas*	Jeon et al., 2004; Mattes et al., 2008
	*Variovorax*	Nogales et al., 2001; Eriksson et al., 2003; Uhlik et al., 2009
γ-Proteobacteria	*Acinetobacter*	Brown and Cooper, 1992; Hedlund et al., 1999; Cappa et al., 2014
	*Pseudomonas*	Whyte et al., 1997; Margesin et al., 2003b; Ma et al., 2006; Field and Sierra-Alvarez, 2008; Haritash and Kaushik, 2009; Harayama and Timmis, 2012; Feng et al., 2014; Parales, 2015
	*Shigella*	Amund et al., 1997
TM7, Saccharibacteria	TM7-1	Militon et al., 2010; Winsley et al., 2014
	TM7-3	Hamamura et al., 2006; Wu et al., 2008; Winsley et al., 2014


In addition to our study, the predominance of Proteobacteria as hydrocarbon degraders has also been observed in other studies. In a recent study by Eriksson et al. (2001, 2003), mixed communities of PAH degraders were enriched from alpine soils under both, aerobic and anaerobic conditions resulting in a few predominant bacterial genera, namely *Pseudomonas*, *Sphingomonas*, and *Variovorax*. The predominance of these three taxa was also determined by Saul et al. (2005) in hydrocarbon-contaminated Antarctic soil. Representatives of β-Proteobacteria, like *Polaromonas* and *Variovorax*, were also identified as PCB degraders in Aroclor-microcosms. *Variovorax* was recently identified in the bacterial community of PCB-polluted soil; and both PCB and PAH degradation of *Variovorax* isolates was described even under aerobic and low temperature conditions (Eriksson et al., 2003). Psychrophilic *Polaromonas* species often isolated from glacial environments like an alpine cryoconite are already known as hydrocarbon-degrading (e.g., naphthalene, dichloroethene) bacteria with optimal features for application in remediation of contaminated cold environments (Mattes et al., 2008; Margesin et al., 2012; Franzetti et al., 2013).

## Conclusion

The present study was conducted with the aim of evaluating both, the level of contamination with the hazardous pollutants PCBs, PAHs, and OCPs as well as the bacterial diversity in a cryoconite on the alpine Jamtalferner glacier in Austria. The obtained results indicate that in carbon-poor environments, like glaciers, the presence of pollutants can strongly force for the selection of strains able to metabolize them. The enrichment of bacteria on PCB droplets confirmed the assumption that pollution causes the stimulation of pollutant-degrading microorganisms (Jordahl et al., 1997; Ding et al., 2009); and thus reflected the level of contamination retrieved in the chemical analyses of the cryoconite. Our results indeed indicate that contaminated glacial areas can be very important reservoirs for bacteria with potential applications in bioremediation of contaminated remote cold areas. Further analyses will be important to elucidate the degradation of more pollutants, e.g., PAHs and PCBs by bacteria, but also by fungi and archaea. Bacterial isolates have to be characterized in more detail concerning their effective degradation capability as well as application in bioremediation processes.

## Author Contributions

RS and NW-B conceived the experiments. NW-B performed all experiments, except HGRC/HRMS analyses performed by K-WS. MF performed bioinformatics and statistical analyses. NW-B and RS wrote the manuscript.

## Conflict of Interest Statement

The authors declare that the research was conducted in the absence of any commercial or financial relationships that could be construed as a potential conflict of interest.
