# Supplementary material for: Polychlorinated Biphenyl (PCB)-Degrading Potential of Microbes Present in a Cryoconite of Jamtalferner Glacier
Source: Front Microbiol. 2017 Jun 15;8:1105. doi: 10.3389/fmicb.2017.01105 (PMC5471330; doi:10.3389/fmicb.2017.01105)
Supplement: Supplementary file 1 [file Data_Sheet_1.DOCX]

Supplementary Material

**Polychlorinated biphenyls (PCB)-degrading potential of microbes present in a cryoconite of Jamtalferner glacier**

Nancy Weiland-Bräuer^1^, Martin A. Fischer^1^, Karl-Werner Schramm^2^ and Ruth A. Schmitz^1^*

*** Correspondence:** Ruth A. Schmitz, [rschmitz@ifam.uni-kiel.de](mailto:rschmitz@ifam.uni-kiel.de)

# Supplementary Figures and Tables

## Supplementary Figures


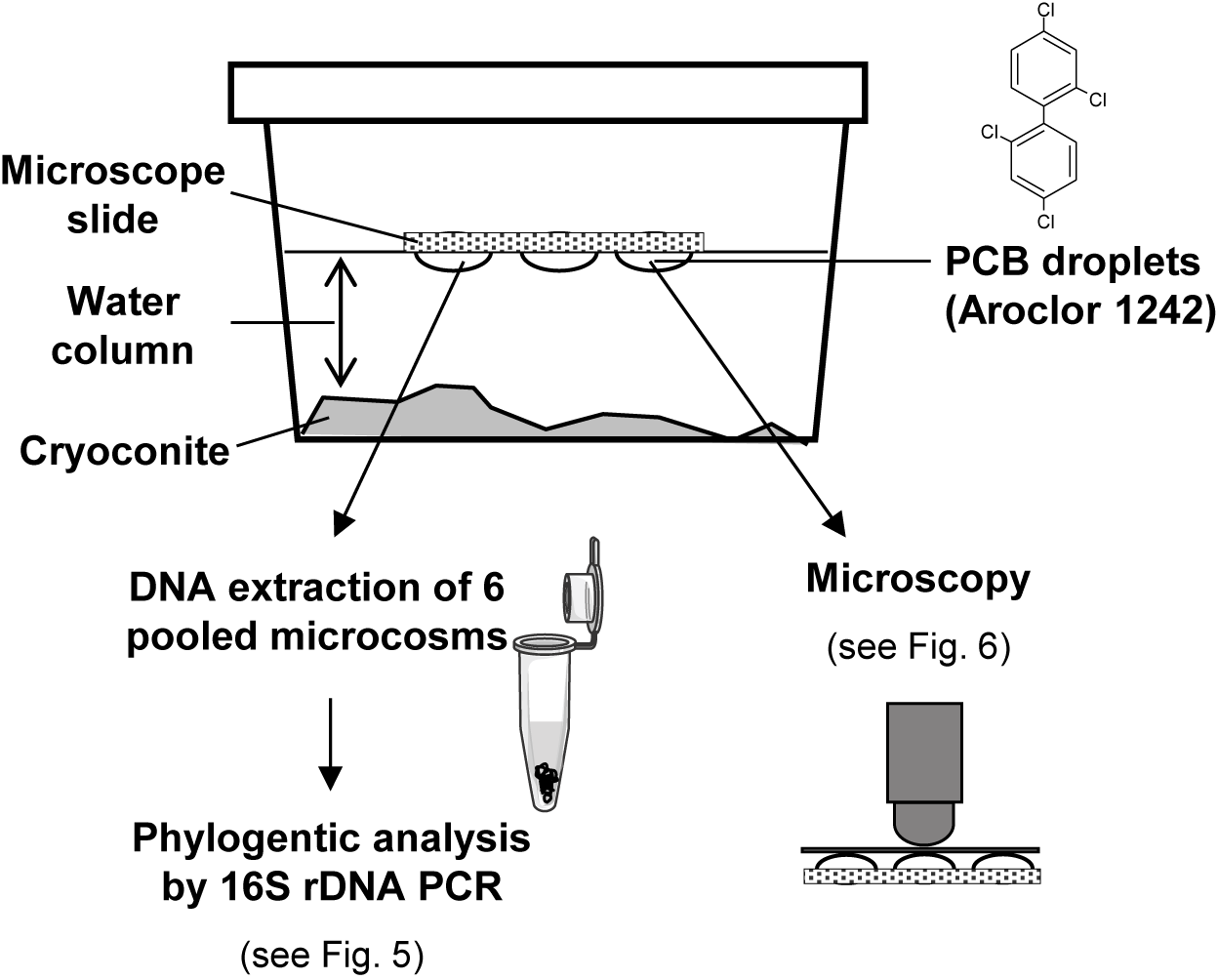


**Fig. S1: Microcosms for biofilm analysis on PCB oil.** 2 mL of the cryoconite sample and 20 mL sterile tap water were placed in a 100 mL plastic vessel. Droplets of 2 µL PCB oil (Aroclor 1242) were placed on microscope slides which were positioned downwards on the water surface. The microcosms were incubated at 4 °C. Three droplets were used for microscopy analysis and 6 droplets were used for DNA extraction (see Materials and Methods) and further phylogenetic analysis by 16S rDNA.

## Supplementary Tables

**Tab. S1: Primer used for amplification of key gene *bphA*.**

| **Target group (species with Accession No.)** | **Primer sequence (5´→ 3´)** | **Melting temperature** |
| --- | --- | --- |
| **universal** | F463: CGC GTS GMW ACC TAC AAR G | 59 |
|  | R674: GGTACATGTCRCTGCAGAAYTGC | 62 |
| **PCB Gram- group 1**  (*Pseudomonas pseudoalacaligenes*, M83673; *Burkholderia cepacia*, M86348; *Pseudomonas sp*., U95054) | F: GTGAAGTGGGTTACCAATTGGA | 64 |
|  | R: GGCGATATTCTTCCTTGATCTC | 64 |
| **PCB Gram- group 2** (*Burkholderia sp*., AJ010057; *Comamonas testosteroni*, U47638) | F: TTAGGTGGTCCCGCAACTGGA | 66 |
|  | R: AGCGGAATTCCTCCTTGATATC | 64 |
| **PCB Gram- group 3** (*Pseudomonas sp*., D17319; *Ralstonia eutropha*, X97923) | F: TGACGTTCAAGCGTCGCTGGA | 66 |
|  | R: GCGGAACTCTTCCTTGATGTC | 64 |
| **PCB Rhodo group 1** (*Rhodococcus erthyropolis*, D88020; *Rhodococcus sp*., U27591; *Rhodococcus globerulus*, X80041) | F: TCAATTGGGTCGCACCTCAAC | 64 |
|  | R: CCTGTACTCCTCCTTGATCTC | 64 |
| **PCB Rhodo group 2** (*Rhodococcus sp*., D32142; *Rhodococcus erthyropolis*, U24277) | F: TGACTGACGTGCAATGTGAACC | 66 |
|  | R: GGCGGTATTCCTCTTTCATCTC | 66 |

**Tab. S2: Accession numbers of bacterial isolates enriched in Nutrient Broth and on PCB droplets.**

| **Isolate designation** | **Enrichment medium** | **Accession No.** |
| --- | --- | --- |
| G4.1 | Nutrient Broth | KT924431 |
| G20.1 | Nutrient Broth | KT924432 |
| G20.2 | Nutrient Broth | KT924433 |
| G30.1 | Nutrient Broth | KT924434 |
| G30.2 | Nutrient Broth | KT924435 |
| G37.1 | Nutrient Broth | KT924436 |
| G37.2 | Nutrient Broth | KT924437 |
| G37.3 | Nutrient Broth | KT924438 |
| G37.4 | Nutrient Broth | KT924439 |
| PCB1 | PCB droplets | KT931670 |
| PCB2 | PCB droplets | KT931671 |
| PCB3 | PCB droplets | KT931672 |
| PCB4 | PCB droplets | KT931673 |
| PCB8 | PCB droplets | KT931674 |
| PCB9 | PCB droplets | KT931675 |
| PCB10 | PCB droplets | KT931676 |
| PCB12 | PCB droplets | KT931677 |
| PCB14 | PCB droplets | KT931678 |
| PCB25 | PCB droplets | KT931679 |
| PCB27 | PCB droplets | KT931680 |
| PCB28 | PCB droplets | KT931681 |
| PCB29 | PCB droplets | KT931682 |
| PCB30 | PCB droplets | KT931683 |
| PCB31 | PCB droplets | KT931684 |
| PCB32 | PCB droplets | KT931685 |
| PCB33 | PCB droplets | KT931686 |
| PCB34 | PCB droplets | KT931687 |
| PCB35 | PCB droplets | KT931688 |
| PCB36 | PCB droplets | KT931689 |
| PCB37 | PCB droplets | KT931690 |
| PCB38 | PCB droplets | KT931691 |
| PCB40 | PCB droplets | KT931692 |
| PCB41 | PCB droplets | KT931693 |
| PCB43 | PCB droplets | KT931694 |
| PCB45 | PCB droplets | KT931695 |
| PCB46 | PCB droplets | KT931696 |
| PCB49 | PCB droplets | KT931697 |
| PCB50 | PCB droplets | KT931698 |
| PCB52 | PCB droplets | KT931699 |
| PCB53 | PCB droplets | KT931700 |
| PCB54 | PCB droplets | KT931701 |
| PCB55 | PCB droplets | KT931702 |
| PCB56 | PCB droplets | KT931703 |
| PCB59 | PCB droplets | KT931704 |
| PCB61 | PCB droplets | KT931705 |
| PCB62 | PCB droplets | KT931706 |

**Tab. S3: Isotopically labeled quantification standards for HRGC/HRMS analyses.**

| PCB (^13^C_12)_ | PCB #28, PCB #52, PCB #77, PCB #81, PCB #101, PCB #105, PCB #114, PCB #118, PCB #123, PCB #126, PCB #138, PCB #153, PCB #156, PCB #157, PCB #167, PCB #169, PCB #180, PCB #189 |
| --- | --- |
| PAH | Napthalene-D8, Acenaphthylene-D8, Acenaphthene-D10, Fluorene-D10, Phenanthene-D10, Anthracene-D10, Fluoranthene-D10, Pyrene-D10, Benzo(a)anthracene-D12, Chrysene-D12, Benzo(b)fluoranthene-D12, Benzo(k)fluoranthene-D12, Benzo(a)pyrene-D12, Indeno(1,2,3-c,d)pyrene-D12, Benzo(g,h,i)- perylene-D12, Dibenzo(a,h)anthracene-D14 |
| OCP | Pentachlorobenzene ^13^C_6_, α-HCH ^13^C_6_, γ-HCH ^13^C_6_, β-HCH ^13^C_6_, δ-HCH ^13^C_6_, Pentachloroanisole ^13^C_6_, Hexachlorobenzene ^13^C_6_, Heptachlor ^13^C_10_, Aldrin ^13^C_12_, Octachlorostyrene ^13^C_6_, oxy-Chlordane ^13^C_10_, Heptachloroepoxide ^13^C_10_, 2,4’-DDE ^13^C_12_, 4,4’-DDE ^13^C_12_, trans-Chlordane ^13^C_12_, Endosulfan-I ^13^C_9_, Endosulfan-II ^13^C_9_, Endosulfan sulphate ^13^C_9_, 4,4’-DDD D_8_, Dieldrin ^13^C_12_, 2,4’-DDT ^13^C_12_, 4,4’-DDT ^13^C_12_, Methoxychlor ^13^C_12_, Mirex ^13^C_10_ |

**Tab. S4: Recovery standards for HRGC/HRMS analyses.**

| PCB | ^13^C_12_-2,3',4’,5-Tetrachlorobiphenyl, ^13^C_12_-2,3,3’,5,5’-Pentachlorobiphenyl, ^13^C_12_-2,2',3,3’,4,4',5-Heptachlorobiphenyl |
| --- | --- |
| PAH | Pentachlorotoluene (PCT), ^13^C_12_-1,2,3,7,8,9-Hexachlorodibenzo-p-dioxin |
| OCP | Pentachlorotoluene (PCT), ^13^C_12_-1,2,3,7,8,9-Hexachlorodibenzo-p-dioxin |

**Tab. S5: Parameters for analytical measurement of PCBs, PAHs and OCPs in the cryoconite sample using HRGC/HRMS.**

|  | **GC parameters** | **MS parameters** |
| --- | --- | --- |
| **PCB** | Type: Agilent 5890 Series II  Column: Stx-CLPesticides2, length 30 m, ID 0,25 mm, ft 0.2 µm (Restek, Bad Homburg, Germany)  Temperature program: 90 °C, 1.5 min, 20 °C min^-1^, 170 °C, 7.5 min, 3.5 °C min^-1^, 285 °C, 20 °C min^-1^, 320 °C, 10 min  Carrier gas: Helium, head pressure: 16 psi  Injector: Cooled injection system KAS4 (Gerstel, Mühlheim an der Ruhr, Germany)  Temperature program injector: 120 °C, 12 °C s^-1^, 280 °C, 5 min  Temperature transferline: 300 °C  Auto sampler: A200S (CTC)  Injection volume: 0.7 µL splitless | Type: MAT 95S (Thermo Fisher Scientific, Darmstadt, Germany)  Ionization mode: EI+, 47 eV, 260 °C  Resolution: > 9000  Detection: SIM mode |
| **PAH** | Type: Agilent 5890 Series II;  Column: Rtx-CLPesticides 2, 30 m, 0.25 mm ID, 0.2 µm film thickness (Restek, Bad Homburg, Germany)  Temperature program: 60 °C, 1.5 min, 10 °C min^-1^, 225 °C, 5 °C min^-1^, 290 °C, 15 °C min^-1^, 315 °C, 20 min;  Carrier gas: Helium, head pressure: 16 psi  Injector: Cooled injection system KAS 3 (Gerstel, Mühlheim an der Ruhr, Germany)  Temperature program injector: 120 °C, 12 °C s^-1^, 280 °C, 5 min;  Temperature transferline: 300 °C  Injektion volume: 0.5 µL splitless | Type: MAT 95 (Thermo Fisher Scientific, Darmstadt, Germany)  Ionization mode: EI+, 47 eV, 260 °C Resolution: > 8000;  Detection: SIM mode  Calibration substance: FC-5311 |
| **OCP** | Type: Agilent 6890  Column: Rtx-Dioxin2, length 40 m, ID 0.18 mm, ft 0.18 µm (Restek, Bad Homburg, Germany)  Temperature program: 60 °C, 1.5 min, 25 °C min^-1^, 140 °C, 8 °C min^-1^, 300 °C, 20 min  Carrier gass: Helium, 1.3 mL min^-1^  Injector: Cooled injection system KAS4 (Gerstel, Mühlheim an der Ruhr, Germany)  Temperature program injector: 120 °C, 12 °C s^-1^, 280 °C, 5 min  Temperature transferline: 300 °C  Autosampler: A200S (CTC)  Injection volume: 0.5 µL splitless | Type: MAT 95XL (Thermo Fisher Scientific, Darmstadt, Germany)  Ionization mode: EI, 50 eV, 260 °C  Resolution: > 9000  Detection: SIM mode |

**Tab. S6: Identified OTUs in the cryoconite from Jamtalferner glacier with corresponding taxonomic classification (in order of sequence abundance).**

| **OTU** | **Taxonomy** | | | | |
| --- | --- | --- | --- | --- | --- |
|  | **Phylum** | **Class** | **Order** | **Family** | **Genus** |
| Otu001 | Proteobacteria | α-Proteobacteria | unclassified | unclassified | unclassified |
| Otu002 | Cyanobacteria | Cyanobacteria | Family II | GpIIa | unclassified |
| Otu003 | Proteobacteria | γ-Proteobacteria | Pseudomonadales | Pseudomonadaceae | *Pseudomonas* |
| Otu004 | Proteobacteria | β-Proteobacteria | Burkholderiales | Alcaligenaceae | *Achromobacter* |
| Otu005 | Firmicutes | Clostridia | Clostridiales | Clostridiaceae | *Clostridium* |
| Otu006 | Proteobacteria | γ-Proteobacteria | Pseudomonadales | Moraxellaceae | *Acinetobacter* |
| Otu007 | Proteobacteria | α-Proteobacteria | Sphingomonadales | Sphingomonadaceae | *Sphingomonas* |
| Otu008 | Proteobacteria | α-Proteobacteria | Rhodospirillales | Acetobacteraceae | *Roseomonas* |
| Otu009 | Proteobacteria | α-Proteobacteria | SAR11 | Candidatus Pelagibacter | unclassified |
| Otu010 | Firmicutes | Clostridia | Clostridiales | Peptococcaceae | *Desulfosporosinus* |
| Otu011 | Bacteroidetes | Cytophagia | Cytophagales | Cytophagaceae | *Hymenobacter* |
| Otu012 | Candidatus Saccharibacteria | unclassified | unclassified | unclassified | unclassified |
| Otu013 | unclassified | unclassified | unclassified | unclassified | unclassified |
| Otu014 | Proteobacteria | γ-Proteobacteria | Pseudomonadales | Pseudomonadaceae | *Pseudomonas* |
| Otu015 | Firmicutes | Bacilli | Bacillales | Staphylococcaceae | *Staphylococcus* |
| Otu016 | Proteobacteria | α-Proteobacteria | Rhizobiales | unclassified | unclassified |
| Otu017 | Bacteroidetes | unclassified | unclassified | unclassified | unclassified |
| Otu018 | Bacteroidetes | unclassified | unclassified | unclassified | unclassified |
| Otu019 | Bacteroidetes | Sphingobacteriia | Sphingobacteriales | Chitinophagaceae | unclassified |
| Otu020 | Proteobacteria | α-Proteobacteria | Rhizobiales | Bradyrhizobiaceae | unclassified |
| Otu021 | Bacteroidetes | Sphingobacteriia | Sphingobacteriales | Chitinophagaceae | unclassified |
| Otu022 | Firmicutes | Clostridia | Clostridiales | Clostridiaceae | *Clostridium* |
| Otu023 | Proteobacteria | α-Proteobacteria | Rhodospirillales | Acetobacteraceae | *Roseomonas* |
| Otu024 | Cyanobacteria | Cyanobacteria | Family II | GpIIa | unclassified |
| Otu025 | Proteobacteria | α-Proteobacteria | unclassified | unclassified | unclassified |
| Otu026 | Proteobacteria | α-Proteobacteria | unclassified | unclassified | unclassified |
| Otu027 | Proteobacteria | α-Proteobacteria | Caulobacterales | Caulobacteraceae | *Phenylobacterium* |
| Otu028 | Candidatus Saccharibacteria | unclassified | unclassified | unclassified | unclassified |
| Otu029 | Proteobacteria | α-Proteobacteria | unclassified | unclassified | unclassified |
| Otu030 | Actinobacteria | Actinobacteria | Actinomycetales | unclassified | unclassified |
| Otu031 | Proteobacteria | α-Proteobacteria | unclassified | unclassified | unclassified |
| Otu032 | Proteobacteria | β-Proteobacteria | Burkholderiales | Alcaligenaceae | *Achromobacter* |
| Otu033 | Proteobacteria | α-Proteobacteria | unclassified | unclassified | unclassified |
| Otu034 | Proteobacteria | α-Proteobacteria | unclassified | unclassified | unclassified |
| Otu035 | Bacteroidetes | Sphingobacteriia | Sphingobacteriales | unclassified | unclassified |
| Otu036 | Firmicutes | Clostridia | Clostridiales | Clostridiaceae | *Clostridium* |
| Otu037 | Firmicutes | unclassified | unclassified | unclassified | unclassified |
| Otu038 | Candidatus Saccharibacteria | unclassified | unclassified | unclassified | unclassified |
| Otu039 | Proteobacteria | α-Proteobacteria | unclassified | unclassified | unclassified |
| Otu040 | Chloroflexi | Anaerolineae | Anaerolineales | Anaerolineaceae | unclassified |
| Otu041 | Acidobacteria | Acidobacteria Gp3 | Gp3 | unclassified | unclassified |
| Otu042 | Cyanobacteria | Cyanobacteria | Family II | GpIIa | unclassified |
| Otu043 | Actinobacteria | Actinobacteria | Actinomycetales | unclassified | unclassified |
| Otu044 | Proteobacteria | γ-Proteobacteria | Xanthomonadales | Xanthomonadaceae | *Rhodanobacter* |
| Otu045 | Bacteroidetes | Sphingobacteriia | Sphingobacteriales | Chitinophagaceae | *Sediminibacterium* |
| Otu046 | Proteobacteria | γ-Proteobacteria | Pseudomonadales | Pseudomonadaceae | *Pseudomonas* |
| Otu047 | Firmicutes | Clostridia | Clostridiales | Clostridiaceae | unclassified |
| Otu048 | Chloroflexi | Anaerolineae | Anaerolineales | Anaerolineaceae | *Levilinea* |
| Otu049 | Proteobacteria | α-Proteobacteria | unclassified | unclassified | unclassified |
| Otu050 | Proteobacteria | α-Proteobacteria | unclassified | unclassified | unclassified |
| Otu051 | Gemmatimonadetes | Gemmatimonadetes | Gemmatimonadales | Gemmatimonadaceae | *Gemmatimonas* |
| Otu052 | Acidobacteria | Acidobacteria Gp1 | Granulicella | unclassified | unclassified |
| Otu053 | unclassified | unclassified | unclassified | unclassified | unclassified |
| Otu054 | Cyanobacteria | Cyanobacteria | Family II | GpIIa | unclassified |
| Otu055 | Proteobacteria | α-Proteobacteria | unclassified | unclassified | unclassified |
| Otu056 | Proteobacteria | γ-Proteobacteria | Pseudomonadales | Moraxellaceae | *Acinetobacter* |
| Otu057 | Proteobacteria | γ-Proteobacteria | Pseudomonadales | Moraxellaceae | *Enhydrobacter* |
| Otu058 | Proteobacteria | δ-Proteobacteria | Myxococcales | Kofleriaceae | *Kofleria* |
| Otu059 | Proteobacteria | γ-Proteobacteria | Pseudomonadales | Moraxellaceae | *Acinetobacter* |
| Otu060 | Proteobacteria | α-Proteobacteria | unclassified | unclassified | unclassified |
| Otu061 | Proteobacteria | β-Proteobacteria | Burkholderiales | Comamonadaceae | unclassified |
| Otu062 | Bacteroidetes | unclassified | unclassified | unclassified | unclassified |
| Otu063 | Planctomycetes | Planctomycetia | Planctomycetales | Planctomycetaceae | *Aquisphaera* |
| Otu064 | unclassified | unclassified | unclassified | unclassified | unclassified |
| Otu065 | Actinobacteria | Actinobacteria | Actinomycetales | unclassified | unclassified |
| Otu066 | Cyanobacteria | Chloroplast | Chloroplast | Streptophyta | unclassified |
| Otu067 | Firmicutes | Clostridia | Clostridiales | Clostridiales Incertae Sedis XI | *Finegoldia* |
| Otu068 | Proteobacteria | α-Proteobacteria | Sphingomonadales | Erythrobacteraceae | *Erythrobacter* |
| Otu069 | Candidatus Saccharibacteria | unclassified | unclassified | unclassified | unclassified |
| Otu070 | Proteobacteria | α-Proteobacteria | unclassified | unclassified | unclassified |
| Otu071 | Proteobacteria | α-Proteobacteria | unclassified | unclassified | unclassified |
| Otu072 | Proteobacteria | β-Proteobacteria | Burkholderiales | Alcaligenaceae | *Achromobacter* |
| Otu073 | Firmicutes | Bacilli | Bacillales | unclassified | unclassified |
| Otu074 | Proteobacteria | α-Proteobacteria | Rhodobacterales | Rhodobacteraceae | *Rhodobacter* |
| Otu075 | Firmicutes | Clostridia | Clostridiales | Clostridiaceae | *Clostridium* |
| Otu076 | unclassified | unclassified | unclassified | unclassified | unclassified |
| Otu077 | unclassified | unclassified | unclassified | unclassified | unclassified |
| Otu078 | Firmicutes | Clostridia | Clostridiales | Ruminococcaceae | *Clostridium* |
| Otu079 | Bacteroidetes | Bacteroidia | Bacteroidales | Porphyromonadaceae | *Paludibacter* |
| Otu080 | Proteobacteria | δ-Proteobacteria | Syntrophobacterales | Syntrophaceae | *Smithella* |
| Otu081 | Proteobacteria | α-Proteobacteria | unclassified | unclassified | unclassified |
| Otu082 | Bacteroidetes | unclassified | unclassified | unclassified | unclassified |
| Otu083 | Proteobacteria | β-Proteobacteria | Burkholderiales | Alcaligenaceae | *Achromobacter* |
| Otu084 | unclassified | unclassified | unclassified | unclassified | unclassified |
| Otu085 | Fusobacteria | Fusobacteriia | Fusobacteriales | Leptotrichiaceae | *Leptotrichia* |
| Otu086 | Proteobacteria | α-Proteobacteria | unclassified | unclassified | unclassified |
| Otu087 | Proteobacteria | α-Proteobacteria | unclassified | unclassified | unclassified |
| Otu088 | Bacteroidetes | Cytophagia | Cytophagales | Cytophagaceae | unclassified |
| Otu089 | Proteobacteria | α-Proteobacteria | unclassified | unclassified | unclassified |
| Otu090 | Actinobacteria | Actinobacteria | Actinomycetales | unclassified | unclassified |
| Otu091 | Planctomycetes | Planctomycetia | Planctomycetales | Planctomycetaceae | *Aquisphaera* |
| Otu092 | Bacteroidetes | Sphingobacteriia | Sphingobacteriales | Sphingobacteriaceae | unclassified |
| Otu093 | Bacteroidetes | unclassified | unclassified | unclassified | unclassified |
| Otu094 | Cyanobacteria | Cyanobacteria | Family II | GpIIa | unclassified |
| Otu095 | Actinobacteria | Actinobacteria | Actinomycetales | Actinomycetaceae | *Actinomyces* |
| Otu096 | Acidobacteria | Acidobacteria Gp4 | Blastocatella | unclassified | unclassified |
| Otu097 | Firmicutes | Clostridia | Clostridiales | unclassified | unclassified |
| Otu098 | Firmicutes | Bacilli | Bacillales | Bacillales Incertae Sedis XI | *Gemella* |
| Otu099 | Bacteroidetes | unclassified | unclassified | unclassified | unclassified |
| Otu100 | Firmicutes | Bacilli | Lactobacillales | Streptococcaceae | *Streptococcus* |
| Otu101 | Bacteroidetes | Sphingobacteriia | Sphingobacteriales | Chitinophagaceae | *Chitinophaga* |
| Otu102 | Cyanobacteria | Cyanobacteria | Family II | GpIIa | unclassified |
| Otu103 | Proteobacteria | unclassified | unclassified | unclassified | unclassified |
| Otu104 | Proteobacteria | α-Proteobacteria | unclassified | unclassified | unclassified |
| Otu105 | Proteobacteria | γ-Proteobacteria | Xanthomonadales | Xanthomonadaceae | unclassified |
| Otu106 | Proteobacteria | α-Proteobacteria | unclassified | unclassified | unclassified |
| Otu107 | Microgenomates | unclassified | unclassified | unclassified | unclassified |
| Otu108 | Firmicutes | Bacilli | Lactobacillales | Streptococcaceae | *Streptococcus* |
| Otu109 | Actinobacteria | Actinobacteria | Actinomycetales | Microbacteriaceae | unclassified |
| Otu110 | Proteobacteria | α-Proteobacteria | unclassified | unclassified | unclassified |
| Otu111 | Bacteroidetes | Sphingobacteriia | Sphingobacteriales | Chitinophagaceae | unclassified |
| Otu112 | Firmicutes | Clostridia | Clostridiales | unclassified | unclassified |
| Otu113 | Cyanobacteria | Cyanobacteria | Family II | GpIIa | unclassified |
| Otu114 | unclassified | unclassified | unclassified | unclassified | unclassified |
| Otu115 | Cyanobacteria | Cyanobacteria | Family II | GpIIa | unclassified |
| Otu116 | Firmicutes | unclassified | unclassified | unclassified | unclassified |
| Otu117 | Firmicutes | Bacilli | Lactobacillales | Streptococcaceae | *Lactococcus* |
| Otu118 | Proteobacteria | α-Proteobacteria | unclassified | unclassified | unclassified |
| Otu119 | Actinobacteria | Actinobacteria | unclassified | unclassified | unclassified |
| Otu120 | Proteobacteria | α-Proteobacteria | unclassified | unclassified | unclassified |
| Otu121 | Cyanobacteria | Cyanobacteria | Family II | GpIIa | unclassified |
| Otu122 | Gemmatimonadetes | Gemmatimonadetes | Gemmatimonadales | Gemmatimonadaceae | *Gemmatimonas* |
| Otu123 | Cyanobacteria | Cyanobacteria | Family II | GpIIa | unclassified |
| Otu124 | Proteobacteria | α-Proteobacteria | unclassified | unclassified | unclassified |
| Otu125 | Cyanobacteria | Cyanobacteria | Family II | GpIIa | unclassified |
| Otu126 | Proteobacteria | α-Proteobacteria | unclassified | unclassified | unclassified |
| Otu127 | Proteobacteria | α-Proteobacteria | unclassified | unclassified | unclassified |
| Otu128 | Proteobacteria | α-Proteobacteria | unclassified | unclassified | unclassified |
| Otu129 | Cyanobacteria | Cyanobacteria | Family II | GpIIa | unclassified |
| Otu130 | Cyanobacteria | Cyanobacteria | Family II | GpIIa | unclassified |
| Otu131 | Proteobacteria | α-Proteobacteria | unclassified | unclassified | unclassified |
| Otu132 | unclassified | unclassified | unclassified | unclassified | unclassified |
| Otu133 | unclassified | unclassified | unclassified | unclassified | unclassified |
| Otu134 | Firmicutes | Bacilli | Bacillales | Staphylococcaceae | *Jeotgalicoccus* |
| Otu135 | Proteobacteria | β-Proteobacteria | Burkholderiales | Alcaligenaceae | *Achromobacter* |
| Otu136 | Proteobacteria | γ-Proteobacteria | Pseudomonadales | Moraxellaceae | *Acinetobacter* |
| Otu137 | Acidobacteria | Acidobacteria Gp3 | unclassified | unclassified | unclassified |
| Otu138 | unclassified | unclassified | unclassified | unclassified | unclassified |
| Otu139 | unclassified | unclassified | unclassified | unclassified | unclassified |
| Otu140 | Bacteroidetes | Cytophagia | Cytophagales | Cytophagaceae | unclassified |
| Otu141 | Actinobacteria | Actinobacteria | Actinomycetales | Microbacteriaceae | *Leifsonia* |
| Otu142 | Chloroflexi | Anaerolineae | Anaerolineales | Anaerolineaceae | *Levilinea* |
| Otu143 | Proteobacteria | α-Proteobacteria | Caulobacterales | Caulobacteraceae | unclassified |
| Otu144 | Proteobacteria | γ-Proteobacteria | unclassified | unclassified | unclassified |
| Otu145 | Proteobacteria | β-Proteobacteria | Burkholderiales | Alcaligenaceae | *Achromobacter* |
| Otu146 | Proteobacteria | α-Proteobacteria | unclassified | unclassified | unclassified |
| Otu147 | Firmicutes | Bacilli | Lactobacillales | Lactobacillaceae | *Lactobacillus* |
| Otu148 | Candidatus Saccharibacteria | unclassified | unclassified | unclassified | unclassified |
| Otu149 | unclassified | unclassified | unclassified | unclassified | unclassified |
| Otu150 | Bacteroidetes | unclassified | unclassified | unclassified | unclassified |
| Otu151 | Firmicutes | Clostridia | Clostridiales | unclassified | unclassified |
| Otu152 | Proteobacteria | β-Proteobacteria | Burkholderiales | Comamonadaceae | unclassified |
| Otu153 | Firmicutes | Clostridia | Clostridiales | Peptococcaceae | *Desulfosporosinus* |
| Otu154 | Bacteroidetes | Sphingobacteriia | Sphingobacteriales | Sphingobacteriaceae | *Mucilaginibacter* |
| Otu155 | Proteobacteria | α-Proteobacteria | unclassified | unclassified | unclassified |
| Otu156 | Proteobacteria | γ-Proteobacteria | Pseudomonadales | Pseudomonadaceae | unclassified |
| Otu157 | Proteobacteria | α-Proteobacteria | unclassified | unclassified | unclassified |
| Otu158 | Proteobacteria | α-Proteobacteria | unclassified | unclassified | unclassified |
| Otu159 | Bacteroidetes | Sphingobacteriia | Sphingobacteriales | Chitinophagaceae | *Chitinophaga* |
| Otu160 | Bacteroidetes | Bacteroidia | Bacteroidales | Prevotellaceae | *Prevotella* |
| Otu161 | Acidobacteria | Acidobacteria Gp1 | Granulicella | unclassified | unclassified |
| Otu162 | Proteobacteria | α-Proteobacteria | Rhizobiales | unclassified | unclassified |
| Otu163 | Proteobacteria | α-Proteobacteria | unclassified | unclassified | unclassified |
| Otu164 | Proteobacteria | β-Proteobacteria | Burkholderiales | Comamonadaceae | unclassified |
| Otu165 | Proteobacteria | δ-Proteobacteria | Bdellovibrionales | Bacteriovoracaceae | *Bacteriovorax* |
| Otu166 | Actinobacteria | Actinobacteria | Actinomycetales | Corynebacteriaceae | Corynebacterium |
| Otu167 | unclassified | unclassified | unclassified | unclassified | unclassified |
| Otu168 | Cyanobacteria | unclassified | unclassified | unclassified | unclassified |
| Otu169 | Firmicutes | unclassified | unclassified | unclassified | unclassified |
| Otu170 | Bacteroidetes | Cytophagia | Cytophagales | unclassified | unclassified |
| Otu171 | Cyanobacteria | Chloroplast | Chloroplast | unclassified | unclassified |
| Otu172 | Firmicutes | Clostridia | Clostridiales | Ruminococcaceae | *Clostridium* |
| Otu173 | Cyanobacteria | Chloroplast | Chloroplast | Chlorophyta | unclassified |
| Otu174 | Bacteroidetes | Bacteroidia | Bacteroidales | Porphyromonadaceae | unclassified |
| Otu175 | Proteobacteria | α-Proteobacteria | Caulobacterales | Caulobacteraceae | *Phenylobacterium* |
| Otu176 | Chloroflexi | Anaerolineae | Anaerolineales | Anaerolineaceae | unclassified |
| Otu177 | Bacteroidetes | Sphingobacteriia | Sphingobacteriales | Sphingobacteriaceae | *Pedobacter* |
| Otu178 | Firmicutes | Clostridia | Clostridiales | Syntrophomonadaceae | unclassified |
| Otu179 | Firmicutes | Bacilli | Bacillales | Bacillaceae | *Bacillus* |
| Otu180 | Firmicutes | Clostridia | Clostridiales | Peptococcaceae | *Desulfosporosinus* |
| Otu181 | unclassified | unclassified | unclassified | unclassified | unclassified |
| Otu182 | Bacteroidetes | unclassified | unclassified | unclassified | unclassified |
| Otu183 | Actinobacteria | Actinobacteria | Actinomycetales | unclassified | unclassified |
| Otu184 | Proteobacteria | δ-Proteobacteria | Myxococcales | Kofleriaceae | *Kofleria* |
| Otu185 | Bacteroidetes | Cytophagia | Cytophagales | Cytophagaceae | *Cytophaga* |
| Otu186 | Proteobacteria | α-Proteobacteria | unclassified | unclassified | unclassified |
| Otu187 | Actinobacteria | Actinobacteria | Actinomycetales | unclassified | unclassified |
| Otu188 | Acidobacteria | Acidobacteria Gp1 | Bryocella | unclassified | unclassified |
| Otu189 | Bacteroidetes | Sphingobacteriia | Sphingobacteriales | Sphingobacteriaceae | *Mucilaginibacter* |
| Otu190 | Actinobacteria | Actinobacteria | Actinomycetales | Propionibacteriaceae | *Propionibacterium* |
| Otu191 | Proteobacteria | β-Proteobacteria | Burkholderiales | Oxalobacteraceae | unclassified |
| Otu192 | Cyanobacteria | Chloroplast | Chloroplast | Streptophyta | unclassified |
| Otu193 | Firmicutes | Negativicutes | Selenomonadales | Veillonellaceae | unclassified |
| Otu194 | unclassified | unclassified | unclassified | unclassified | unclassified |
| Otu195 | Proteobacteria | α-Proteobacteria | unclassified | unclassified | unclassified |
| Otu196 | Proteobacteria | β-Proteobacteria | Burkholderiales | Comamonadaceae | unclassified |
| Otu197 | Actinobacteria | Actinobacteria | Actinomycetales | unclassified | unclassified |
| Otu198 | Bacteroidetes | Flavobacteriia | Flavobacteriales | Flavobacteriaceae | unclassified |
| Otu199 | Proteobacteria | α-Proteobacteria | Rhizobiales | Bradyrhizobiaceae | unclassified |
| Otu200 | Actinobacteria | Actinobacteria | Actinomycetales | unclassified | unclassified |
| Otu201 | Bacteroidetes | Sphingobacteriia | Sphingobacteriales | Chitinophagaceae | unclassified |
| Otu202 | Proteobacteria | δ-Proteobacteria | Bdellovibrionales | Bacteriovoracaceae | *Bacteriovorax* |
| Otu203 | Actinobacteria | Actinobacteria | Actinomycetales | unclassified | unclassified |

**Tab. S7: Identified OTUs in the cryoconite from Jamtalferner glacier matching with cryoconite isolates.** Sequences of OTUs (16S V1-V2 region) matching with sequences of isolated bacteria from NB as well as microcosm experiments (complete 16S rRNA fragment) are listed.

| **OTU** | **Taxonomy** | | | **Matching bacterial isolates** |
| --- | --- | --- | --- | --- |
|  | **Phylum** | **Class** | **Genus** |  |
| Otu003 | Proteobacteria | γ-Proteobacteria | *Pseudomonas* | PCB32 |
| Otu007 | Proteobacteria | α-Proteobacteria | *Sphingomonas* | PCB37 |
| Otu014 | Proteobacteria | γ-Proteobacteria | *Pseudomonas* | PCB38, PCB40 |
| Otu015 | Firmicutes | Bacilli | *Staphylococcus* | G30.2 |
| Otu021 | Bacteroidetes | Sphingobacteriia | unclassified | PCB61 |
| Otu030 | Actinobacteria | Actinobacteria | unclassified | PCB30, PCB31, PCB36, PCB55, PCB59 |
| Otu035 | Bacteroidetes | Sphingobacteriia | unclassified | PCB25 |
| Otu043 | Actinobacteria | Actinobacteria | unclassified | PCB27, PCB29 |
| Otu046 | Proteobacteria | γ-Proteobacteria | *Pseudomonas* | G20.1, G20.2, PCB43, PCB62 |
| Otu061 | Proteobacteria | β-Proteobacteria | unclassified | PCB41 |
| Otu065 | Actinobacteria | Actinobacteria | unclassified | PCB34, PCB53 |
| Otu101 | Bacteroidetes | Sphingobacteriia | *Chitinophaga* | PCB45, PCB52 |
| Otu144 | Proteobacteria | γ-Proteobacteria | unclassified | PCB12, PCB14 |
| Otu156 | Proteobacteria | γ-Proteobacteria | unclassified | PCB2, PCB3, PCB10 |
| Otu159 | Bacteroidetes | Sphingobacteriia | *Chitinophaga* | PCB61 |
| Otu164 | Proteobacteria | β-Proteobacteria | unclassified | PCB56 |
| Otu179 | Firmicutes | Bacilli | *Bacillus* | G37.2 |
| Otu183 | Actinobacteria | Actinobacteria | unclassified | G30.1 |
| Otu187 | Actinobacteria | Actinobacteria | unclassified | G37.1 |
| Otu189 | Bacteroidetes | Sphingobacteriia | *Mucilaginibacter* | PCB46 |
| Otu191 | Proteobacteria | β-Proteobacteria | unclassified | G4.1, PCB33 |
